# Dengue Virus Infection Presenting as Membranoproliferative Glomerulonephritis Type 1

**DOI:** 10.7759/cureus.14294

**Published:** 2021-04-05

**Authors:** Sami Alobaidi, Hamza Bali, Mohammad F Tungekar, Ahmed Akl

**Affiliations:** 1 Department of Medicine, University of Jeddah, Jeddah, SAU; 2 Department of Medicine, Dr Soliman Fakeeh Hospital, Jeddah, SAU; 3 Histopathology, Uranologics Ltd, London, GBR; 4 Department of Medicine, Fakeeh College of Medical Sciences, Jeddah, SAU; 5 Urology and Nephrology Centre, Mansoura University, Mansoura, EGY

**Keywords:** dengue infection, acute kidney injury, haematuria, proteinuria, membranoproliferative glomerulonephritis

## Abstract

The renal complications of dengue virus infection cover a wide spectrum of manifestations from acute kidney injury to glomerular injury with nephritic/nephrotic syndrome. Majority of cases remain symptom free and show full recovery. We present a 61-year-old previously healthy male who developed a pyrexial illness with haemolytic anaemia that was diagnosed on the basis of a positive serological test as a case of dengue fever. He received supportive treatment and showed general recovery except for his renal dysfunction that showed persistent proteinuria at 14 gm/24 hours. A kidney biopsy revealed membranoproliferative glomerulonephritis type 1 (MPGN-l). Complete remission was achieved by steroids and mycophenolate mofetil therapy. We provide convincing biopsy evidence that dengue virus is yet another viral cause of MPGN-l and also document its successful management with mycophenolate mofetil and steroids therapy.

## Introduction

Dengue viral infection (DVI), a mosquito-borne infection caused by virus with four serotypes of the genus flavivirus, has a global footprint [1]. The infection results in a variety of clinical manifestations, from an asymptomatic illness to severe and fatal systemic haemorrhagic disease [1,2]. The risk of severe disease is much higher in secondary rather than primary dengue infection. The involvement of kidneys commonly manifests as acute kidney injury caused by plasma leakage syndrome or myoglobinuria [2,3]. A proportion of cases suffer from haematuria and/or nephrotic range proteinuria, believed to be caused by a ‘glomerulonephritic process’ that has not been fully pathologically characterised [4]. Membranoproliferative glomerulonephritis (or its synonym mesangiocapillary glomerulonephritis) of immune complex type or type I (MPGN-I) is a form of glomerular injury that has established association with hepatitis B and hepatitis C virus infections [5]. We describe a case of DVI who presented with nephrotic range proteinuria and impaired kidney function that was diagnosed by light, immunofluorescence and electron microscopy as MPGN-I. Complete remission of proteinuria with recovery of renal function was achieved over a period of one year. We report this case to document DVI as yet another viral cause of MPGN-I and describe its successful management after the biopsy diagnosis.

## Case presentation

A 61-year-old male was admitted to the local hospital in his home town in the Philippines with high-grade fever and haemolytic anaemia with a history of mosquito bites. Intravenous fluids and medications were initiated. Normal serum creatinine and no proteinuria had been recorded one month back on a routine check-up. A diagnosis of DVI was made based on a positive immunoglobulin (Ig)M test for dengue virus. The patient reported no previous history of DVI. On his return to Saudi Arabia, he presented to our out-patient department with significantly elevated blood pressure with his blood pressure readings at 200/100 mm Hg, generalized anasarca, a painful epigastrium and acute kidney injury with a serum creatinine of 247 µmol/L (that worsened on next day to 265 µmol/L), proteinuria 14 gms/24 hours, total serum cholesterol 7.78 µmol/L and serum albumin 29 gm/L. Autoimmune serology (antinuclear antibody (ANA) and antineutrophil cytoplasmic autoantibodies (ANCA) and tests for hepatitis B virus (HBV), hepatitis C virus (HCV), and human immunodeficiency virus (HIV) were negative. Complement levels were low. Urine analysis showed haematuria and leukocyturia. An ultrasound revealed grade I nephropathy and normal-sized kidneys with no pelvicalyceal lesions.

**Figure 1:**
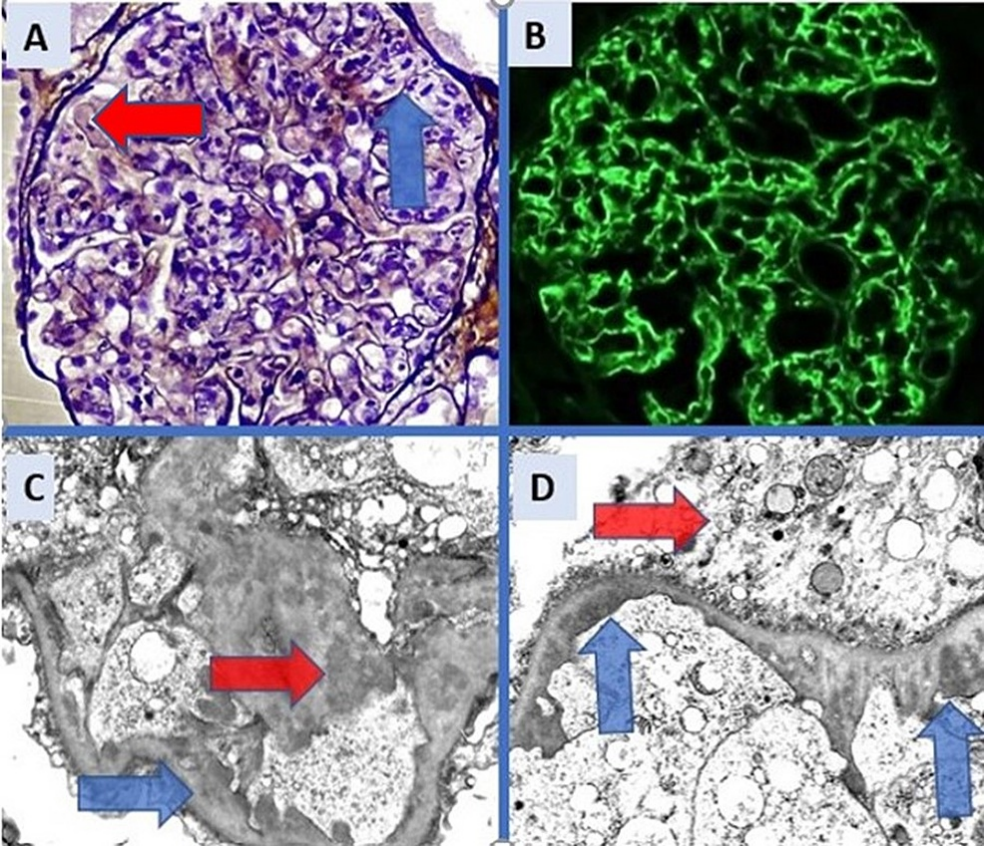
Kidney biopsy A: Glomerulus showing vague lobular profile, intense endocapillary and mesangial hypercellularity, groups of polymorphonuclear leukocytes (blue arrow) and duplication of capillary walls (red arrow). (Jones silver stain, X120), B: Immunofluorescence stain for IgG (X120), C: Electron Micrograph showing subendothelial (blue arrow) and mesangial (red arrow) deposits (X2500), D: Electron micrograph showing more subendothelial deposits (blue arrows) and activated podocyte (red arrow), (X2500).

**Figure 2:**
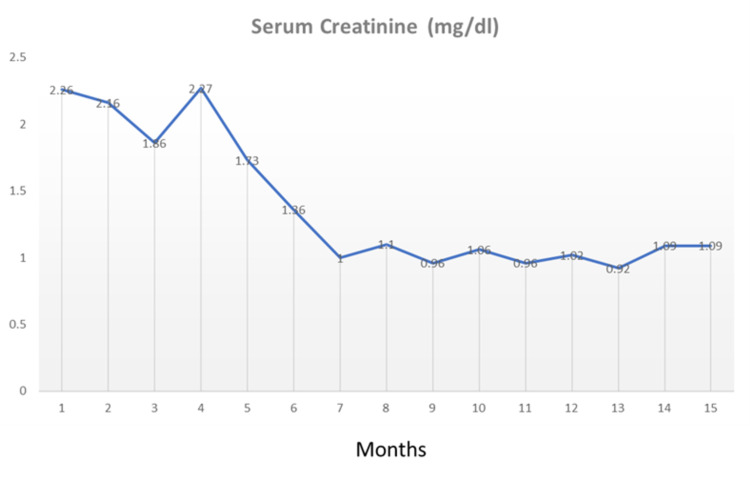
Serum creatinine monitoring through follow up time (months)

**Figure 3:**
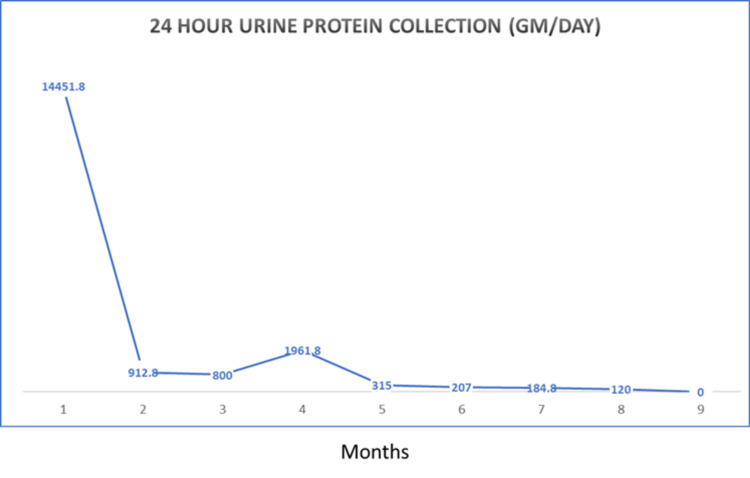
Twenty-four hour urine protein collection monitoring through follow up time (months)

After managing his hypertension and since conservative measures did not produce an improvement in kidney function, a renal biopsy was performed. The biopsy included 16 glomeruli, one of them globally sclerosed (Figure 1). All the perfused glomeruli displayed global endocapillary hypercellularity with lobulated profiles, segmental duplication of basement membranes and polymorphonuclear leukocytes with foci of karyorrhexis (Figure 1A). Mild focal tubulointerstitial scarring affecting less than 5% of the cortex was seen. There was insignificant acute tubular injury and no myoglobin casts. Immunofluorescence microscopy (as seen in Figure 1B) showed capillary wall and mesangial positivity for IgG and C3 (all 3+) and electron microscopy confirmed subendothelial and mesangial electron dense deposits (Figure 1C). These features were diagnostic of MPGN-l (immune complex type). A regimen of high dose steroids (prednisolone 60 mg per day) and mycophenolate mofetil (1 gm twice per day) for one month was decided. A significant improvement in kidney function was achieved with serum creatinine returning to normal (79.6 µmol/L), which was followed by tapering of oral steroids and continuation of mycophenolate mofetil 1 gm twice per day for six months. Proteinuria improved from 14 gm/day to 315 mg/day (then nil) and serum albumin was 4.29 gm/dl after two months of treatment (Figures 2-3). At follow up, 18 months after discharge, the patient was free of oedema and proteinuria with normal serum creatinine levels. He maintained a normal renal profile at a follow up appointment at one year after discharge.

## Discussion

The commonly reported renal involvement in DVI is acute tubular injury due to hypovolaemic shock, direct cytopathic effect of the virus on renal tubules, acute thrombotic microangiopathy or damage caused by myoglobinuria [2]. Rarely acute thrombotic microangiopathy is the cause of renal dysfunction [6]. The glomerular disease with significant proteinuria leading to nephrotic syndrome is also encountered and its pathology often described simply as ‘proliferative glomerulonephritis’ without proper morphological description or classification of the lesion [4]. Some case reports have assumed the presence of glomerular lesions based on findings on urine analysis [7].

The role of immune complexes in causing glomerular injury in human DVI is a subject of debate in the literature although experimental animal studies have confirmed it [8]. Some authors, while acknowledging the formation of immune complexes in DVI, have either ruled out or raised the possibility of immune complex induced glomerular injury in humans [9,10].

Morphologically, MPGN-I is characterised by double contouring of glomerular basement membranes, mesangial proliferation, immune complex (immunoglobulin and complement) deposition in capillary walls and mesangium, that corresponds to subendothelial and mesangial electron dense deposits [11]. MPGN-I is known to be associated with a wide spectrum of aetiologies such as autoimmune disorders and bacterial, viral and parasitic infections, and is well-recognised in hepatitis B and C viral infections [5,11]. The clinical course and management of MPGN-I hinges on identification of the underlying cause [12].

## Conclusions

We describe the first well-documented occurrence of MPGN-I, as a manifestation of immune complex-mediated glomerular injury in a case of DVI that presented with impaired kidney function and significant proteinuria. The patient was satisfactorily managed with appropriate immunosuppression. We emphasize the need for a proper renal biopsy diagnosis in cases of dengue fever presenting with renal dysfunction and proteinuria; the pathological diagnosis prompts appropriate management to achieve rapid clinical improvement and avoid long-term complications.
